# MoS_2_-based absorbers with whole visible spectrum coverage and high efficiency

**DOI:** 10.1038/s41598-022-10280-2

**Published:** 2022-04-15

**Authors:** Mahdieh Hashemi, Narges Ansari, Mahsa Vazayefi

**Affiliations:** 1 Department of Physics, College of Science, Fasa University, Fasa, 74617-81189 Iran; 2 Department of Physics, Faculty of Physics and Chemistry, Alzahra University, Tehran, Iran

**Keywords:** Nanoscience and technology, Nanoscale devices, Nanophotonics and plasmonics

## Abstract

To design highly efficient and broadband nanometer-sized absorbers based on the atomically thin transition metal dichalcogenides (TMDCs), we propose utilizing inclined gold gratings on MoS_2_ monolayer. In the case of gold gratings with zero inclination, coverage of the absorption spectrum in the entire visible range occurs between the values of 42% to 73%. Considerable increase in the absorbed light occurs by introducing 13 nm inclination to the gold gratings with equal values of the grating’s period and width as 60 nm. With the application of this grating, maximum absorption of 88% is reached and the absorption bandwidth covers the entire visible spectrum with only 12% variation of the absorption value relative to this maximum (88%). Footprints of resonant excitation of two different modes in the absorber structure are evident: the named “reflection” mode and localized surface plasmons (LSPs). Inclination of the gratings leads the LSP modes to slide toward the MoS_2_ and causes a remarkable increment in the absorption efficiency. An impressive absorption value of 56% in MoS_2_ monolayer is gained by the gold grating’s inclination of 17 nm. The designed absorber paves a new way in designing TMDC-based absorbers with extended bandwidths and higher efficiencies.

## Introduction

Higher efficiencies of optoelectronic components are directly related to the amount of light which is absorbed in them. Photovoltaic cells, photodetectors, and modulators are few examples of these components which are widely used in the field of green energy production and telecommunication^1–3^. Apart from the absorption quantity, the absorption bandwidth is also a deterministic parameter in absorbers, especially in photovoltaic applications within the visible range^4,5^.

To design miniaturized absorbers and move toward lab-on-chip-size components, two-dimensional (2D) materials together with subwavelength structures on them are proper choices. Among the atomically-thin 2D materials, efficient light absorption occurs in transition metal dichalcogenides (TMDCs) with direct band gaps^6^.

Molybdenum disulfide, MoS_2_, is one of the well-known two-dimensional materials within TMDC’s family with the chemical formula format of $\text {MX}_2$, in which *M* is the symbol of a transition metal element and *X* refers to a chalcogen. In this material, Molybdenum and Sulfur elements are covalently bond in a $S-Mo-S$ form in which the *Mo* atoms are sandwiched between two *S* atoms which are placed in layers with hexagonal arrangement. Layers of MoS_2_ are coupled together by weak van der Waals forces^7^. Bulk of MoS_2_ with indirect band gap of 1.3 ev transforms to a direct band gap semiconductor^8^ in its monolayer state with high ratio of absorption coefficient^9^ and reasonable carrier mobility^10^. Such promising properties of MoS_2_ monolayer makes it attractive for application in hetrostrucrures, memristors, transistors, solar elements, supercapacitors, spintronic devices, and optical elements such as photodetectors^11–17^.

Monolayer of MoS_2_, the 2D material that we focus on it in this paper and has direct band gap in its atomically thin state, absorbs 23%, 6%, and 7% of the incident light at wavelengths of 432 nm, 617 nm, and 664 nm, respectively^18^. Although these absorption amounts are astonishing compared to the atomic thickness of the MoS_2_, to design a MoS_2_-based absorber these values should increase. To do this, using stacks of layers in the form of photonic crystals or quasi-photonic crystals were suggested^4,19–23^. Although within this method broadband absorption efficiencies above 90% is reported, due to the usage of more than hundred layers with multi-stacks of MoS_2_, fabrication of such structure is experimentally complicated.

Utilizing metallic structures in the form of reflecting layers, gratings, and nanoparticles in the absorber structure with their ability in reflecting back the incoming light, supporting surface plasmons (SPs)^24^, or localized surface plasmons (LSPs)^25–27^ are proper choices to enhance light absorption in TMDC-based absorbers^28–31^. SPs, the collective oscillation of electrons, and the LSPs, the trapped electric or magnetic dipoles in the metallic structures, cause enhancement of the electromagnetic field in the nearfield zone which leads to augment of the absorbed light in the structure^32–34^. Even in the case of using uniform metallic layers in the absorber structure in which no SPs or LSPs find the chance of excitation, metallic layers can act like reflecting mirrors which increase the light path length inside the MoS_2_ layer and increase the light absorption^35–38^.

Among MoS_2_-based absorbers that uses metallic layers, broadband light absorption above 80% is reported in the wavelength range of 300 nm to 500 nm which doesn’t cover the full visible spectrum^39,40^. To extend the absorber’s working spectrum, in^30^ a 2D metallic grating is used on MoS_2_ and a photonic crystal was added beneath it. They could extend the working spectrum but at the cost of lowering the absorption efficiency to the amounts around 70% within the wavelength range of 450 nm to 650 nm. As the metallic grating dimensions are set to be subwavelength, when it is illuminated by the incident light, not only the refractive index of the metal affects the light scattering, but also the grating geometry plays an essential role. In a graphene-based photodetector study^35^, it is shown that by inclining the ribbons of gold grating, the excited LSPs will move close to the graphene layer which leads to enhanced absorption in it.

In this paper, we design an absorber which absorbs the incident light in whole visible spectrum, from $\lambda =400$ nm to $\lambda =780$ nm, with high efficiency, maximum absorption value of 88% and not less than 77%. Our proposed absorber structure consists of inclined gold gratings on MoS_2_ monolayer, while the MoS_2_ itself stands on a plane silica substrate. By investigating the origin of the excited modes in the absorber, excitation of two types of modes in the visible spectrum can be distinguished: the named “reflection” modes at shorter wavelengths and LSP modes at longer ones. The “reflection” modes mainly capture the incoming light in the gold grating’s outer face, while the LSP modes excitation happens together with light absorption enhancement in the MoS_2_ layer.

## Absorber Structure

**Figure 1:**
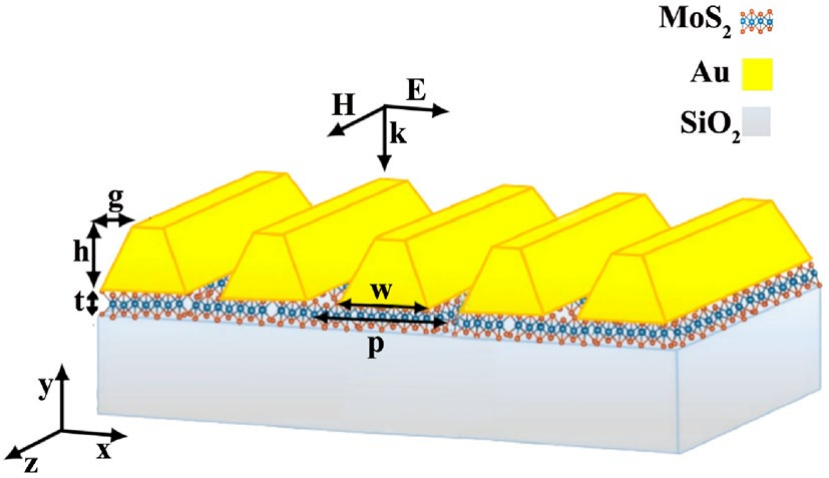
Schematic of our designed absorber with inclined gold gratings on a MoS_2_ monolayer. The *p*, *h*, *w*, and *g* parameters illustrate gold grating’s periodicity, height, width and, inclination, respectively.

Our proposed absorber, as it is shown in Fig. 1, is made up of a uniform MoS_2_ monolayer on a silica substrate with inclined gold ribbons on it. Although we present simulation results in this paper, it has worth mentioning that there are numerous methods to fabricate such 2D gratings with high accuracy. From these methods we can refer e-beam- or soft- lithography, nano-imprinting, plasma assisted surface machinery process, atomic force microscope, laser assisted technique, ion beam writing, and chemical or physical template assisted block copolymer process^41–47^.Thickness of the MoS_2_ layer is indicated by *t* which is set to 0.62 nm throughout the paper^6^. The gold ribbons with height of *h*, width of *w*, and inclination of *g* are infinitely extended along the $z-$ and repeating themselves with periodicity of *p* in $x-$direction (Fig. 1). By sweeping the values of these four geometrical parameters, *p*, *h*, *w*, and *g*, we explore the most efficient absorber design. The incident light impinges the absorber normally in $y-$direction with its magnetic field normal to the incident plane (*xy*-plane). With this transverse magnetic field configuration of the illuminating light relative to the incident plane, it is known as a TM-polarized light.

To calculate the absorbed light value in the designed device finite element method is used and along the $y-$direction the structure terminates by perfect matched layers, while in the $x-$direction periodic boundaries surround its unit cell. To model the complex refractive index of MoS_2_ monolayer the Lorantz model is applied as it is presented in equation (1):

1 $$\begin{aligned} n_{MoS_{2}}=\sqrt{\varepsilon _{\infty }+\sum _{j=1}^{n}{\frac{a_{j}}{\omega _{j}^{2}-\omega ^{2}-i \omega b_{j}}}} \end{aligned}$$

in which $\omega$ and $\varepsilon _{\infty }$ are the incident light frequency and the DC permittivity, respectively. Values of $\omega _{j}$, $a_{j}$, and $b_{j}$ which are the resonant frequency, oscillation power, and damping factor of the $j-$th oscillator, are taken from^18^. Silica refractive index with constant value of 1.5 is selected from^48^. Table of the dispersive complex refractive index of gold that we used in our simulations are from^49^.

## Absorption enhancement by Non-inclined gold gratings

**Figure 2:**
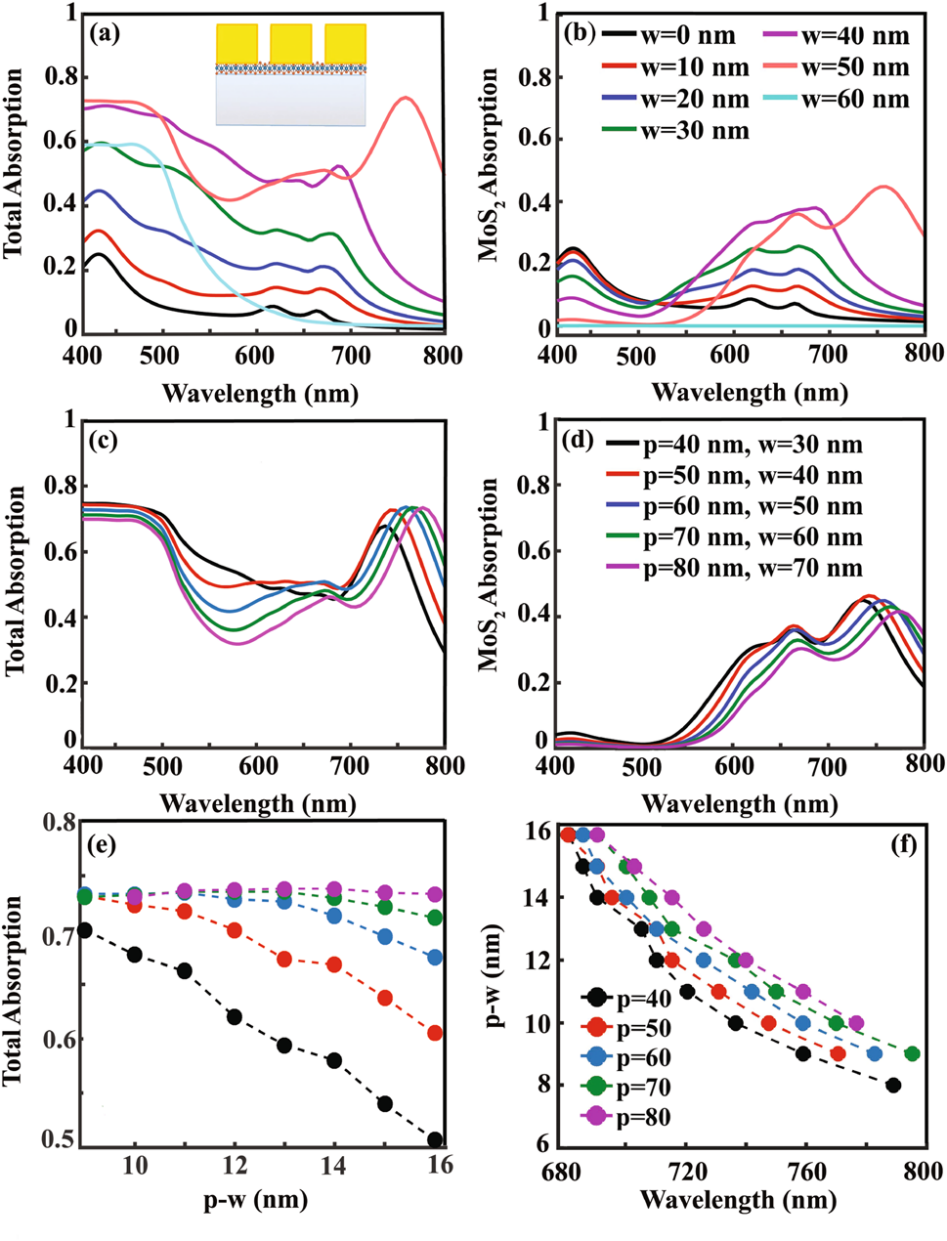
(**a**) Total and (**b**) MoS_2_ absorption when the MoS_2_ is covered by gold grating with $p=60$ nm, $h=110$ nm, and width of ribbons, *w*, varies. Special cases like uniform coverage of MoS_2_ by gold layer ($w=60$ nm, cyan solid line) and no gold grating ($w=0$, black solid line) are also included. (c) total and (d) MoS_2_ absorption when grating pitch varies from $p=40$ nm to $p=80$ nm with *w* of the gratings set for optimum absorption. (e) total absorption of the structure as a function of $p-w$ with different values of *p* (f) $p-w$ versus wavelengths at which maximum total absorption happens for gratings with different periods.

**Figure 3:**
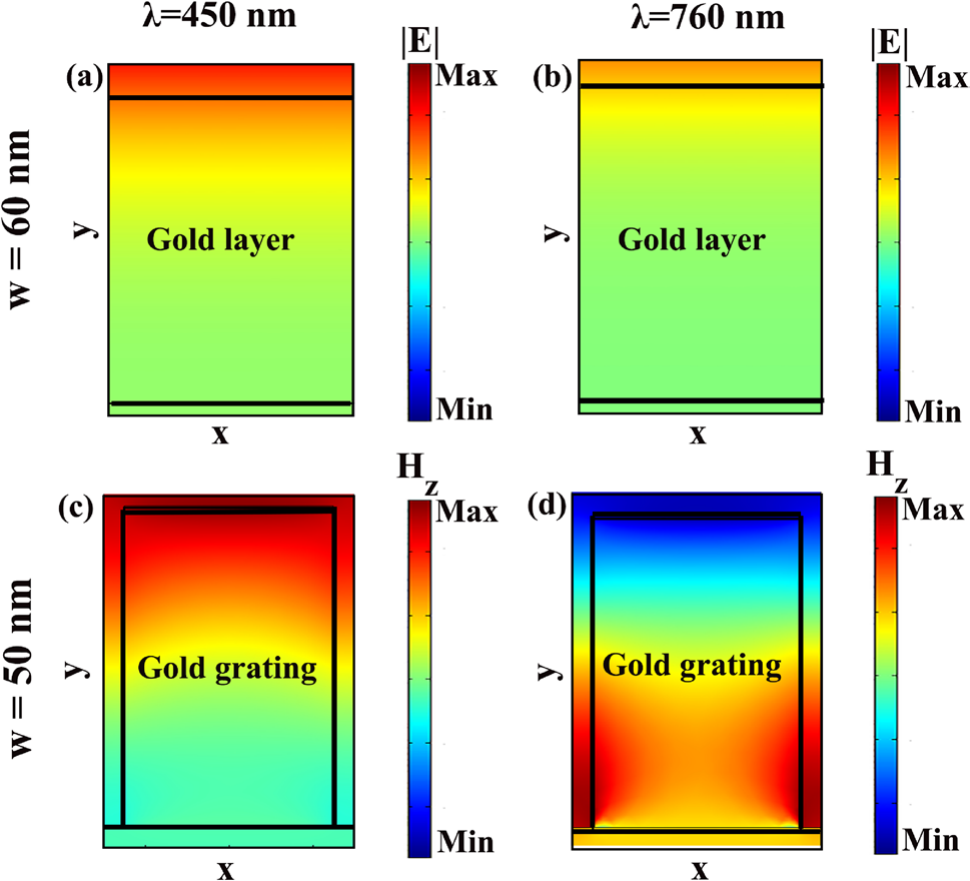
Electric field distribution of the structure when 110 nm-thick uniform gold layer covers the MoS_2_ monolayer at (**a**) $\lambda =450$ nm and (**b**) $\lambda =760$ nm. Distribution of the $z-$component of the magnetic field, $H_{z}$, of the structure when gold grating with $h=110$ nm, $p=60$ nm, and $w=50$ nm covers the MoS_2_ monolayer at (**c**) $\lambda =450$ nm and (**d**) $\lambda =760$ nm.

In our first try, we investigate the effect of utilizing non-inclined gold grating, $g=0$, on MoS_2_. Absorption spectrum of MoS_2_ monolayer on $\text {SiO}_2$ substrate is included in Fig. 2a as a solid black line when the only absorbing layer in the structure is MoS_2_. The three characteristic absorption peaks of MoS_2_ at the wavelengths of 432 nm, 617 nm, and 664 nm which are equivalent to the energies of the direct band gaps of this material, can be clearly recognized. Utilizing gold ribbons on MoS_2_ layer increases both total absorption in the structure (Fig. 2a and c) and in the MoS_2_ layer (Fig. 2b and d). In Fig. 2a and b, *p* and *h* are set as 60 nm and 110 nm, respectively, and the effect of the gold ribbon’s width, *w*, on the total and MoS_2_ absorption is investigated. Increasing the ribbon width up to $w=50~nm$ intensifies the light absorption in a wide range of visible spectrum. If we continue increasing the ribbon’s width in a way to cover fully the MoS_2_ as a uniform gold layer, the absorption spectrum changes to the cyan colored solid line, $w=60$ nm, of Fig. 2a and b. With this uniform gold layer, $w=60$ nm, as it can be seen in Fig. 2a, 60% of the incident light absorbs in the structure within the wavelength range of $\lambda =400$ nm to $\lambda =480$ nm, while in the remaining part of the visible spectrum the absorbed light is negligible. MoS_2_ absorption of this structure, as it is presented in Fig. 2b, illustrates the nearly zero light absorption in MoS_2_ (solid cyan line). To explore the origin of this absorption, in Fig. 3a and b, we include the electric field distribution of the structure at $\lambda =450$ nm with 60% light absorption and at $\lambda =760$ nm with negligible absorption. At $\lambda =450$ nm electric field collection at the outer surface of the gold layer shows enhanced light absorption at the gold surface in a form of a mode that we name it as “reflection”. The origin of this mode excitation is the Ohmic loss of the gold which accumulates the incoming electromagnetic field within a shallow depth of penetration at the gold/air interface. While for longer wavelengths like 760 nm, the incident light scatters back to the free space without any absorption in the structure (Fig. 3b).

Getting back to Fig. 2a and looking at the total light absorption in the structure with $p=60$ nm and $w=50$ nm, with $p-w=10$ nm, the “reflection” mode excitation footprints can be distinguished with 73% light absorption between $\lambda =400$ nm to $\lambda =480$ nm, while another resonant absorption peak at 760 nm is added. To investigate the nature of this enhanced absorption, in Fig. 3c and d, $H_{z}$ distribution at $\lambda =450$ nm and $\lambda =760$ nm are presented (as the incident light polarization is TM, the magnetic field has only *z* component). In Fig. 3c and at the wavelength of 450 nm, the same “reflection” mode as the one that is observed in Fig. 3a can be distinguished, which appears with high intensities at the outer surface of the gold grating. At $\lambda =760$ nm resonant Fabry-Proét-type mode excitation in the grating’s gap can be seen^36,50^. Such Fabry-Proét mode excitation which accompanies resonantly localization of the electromagnetic field in the grating’s gap space is well-known as LSP. The excited LSPs couple to the incident TM polarized light in a way their magnetic field localizes at the bottom of the gap, in the vicinity of MoS_2_ layer, as it is clear in Fig. 3d. Field localization close to the MoS_2_ layer and at the bottom of the grating’s gap, not only intensifies the total absorption in the structure but also enhances light absorption in MoS_2_ to the amount of 45% (Fig. 2b). This 45% value of the light absorption in MoS_2_ monolayer out of the 73% total absorption reveals the significant role of this atomically-thin layer in the absorbed light in the structure.

The occurrence of the two resonant modes, the “reflection” and the LSP modes together in the visible spectrum, prevent minimum absorption of the structure from reaching zero value in the entire range of visible wavelengths. This broadband absorption in the structure with $p=60$ nm and $w=50$ nm keeps the total absorption above 42% in the visible spectrum.

In Fig. 2c and d, gold gratings with different periods, *p*, are applied on MoS_2_. Width of each grating, *w*, are optimized and selected in a way to have optimum absorption. As it can be seen, increasing the grating period from $p=40$ nm to $p=80$ nm doesn’t change the excitation range of the “reflection”-mode, $\lambda =400$ nm to $\lambda =480$ nm, and its absorption efficiency, significantly. Within the period range of $p=50$ nm to $p=80$ nm, the resonant LSP-based absorption peak slightly red shifts, while the peak value doesn’t change significantly and fixes around 73%. These fixed absorption values with grating period changes help manufacturing efficiency to be preserved against grating fabrication inaccuracy.

Excitation of LSPs in the gold grating gaps at the wavelength of the absorption peak turns our attention to the grating’s gap size , $p-w$, role in the absorption efficiency. In Fig. 2e, total absorption of the structure as a function of $p-w$ at the wavelength with maximum absorption is plotted for different grating periods ($p=40$ nm to $p=80$ nm in steps of 10 nm). With the grating’s periods of 60, 70,  and 80 nm, maximum light absorption remains almost constant with nanometer-size-changes of $p-w$ from 9 nm to 14 nm, while for smaller periods like $p=40$ nm the story is totally different. Independence of the maximum light absorption from the minor changes of the grating size (up to 5 nm) is a very good guarantee in experiment to get the simulated results. In a survey of the wavelength with maximum light absorption in structures with different periods as a function of the grating gap’s size, $p-w$, we included the results in Fig. 2f. From Fig. 2f, it can be deduce that, maximum light absorption occurs at longer wavelengthes with smaller values of $p-w$. This way, to set the working wavelength of our absorber in the visible range, acceptable $p-w$ values should be selected between 8 nm to 16 nm.

Up to now, by employing the non-inclined gold gratings on MoS_2_ monolayer, we reached maximum 73% of the total light absorption in the structure and 45% of MoS_2_ absorption, with grating parameters of $h=110$ nm, $p=60$ nm to $p=80$ nm , and $p-w=9$ nm to $p-w=16$ nm. The absorber with $p=60$ nm in which the absorption remains above 42% in the entire visible spectrum, is the structure that we select for further studies in the next section and improving the light absorption in it while keeping its wide band absorption unaffected.

**Figure 4:**
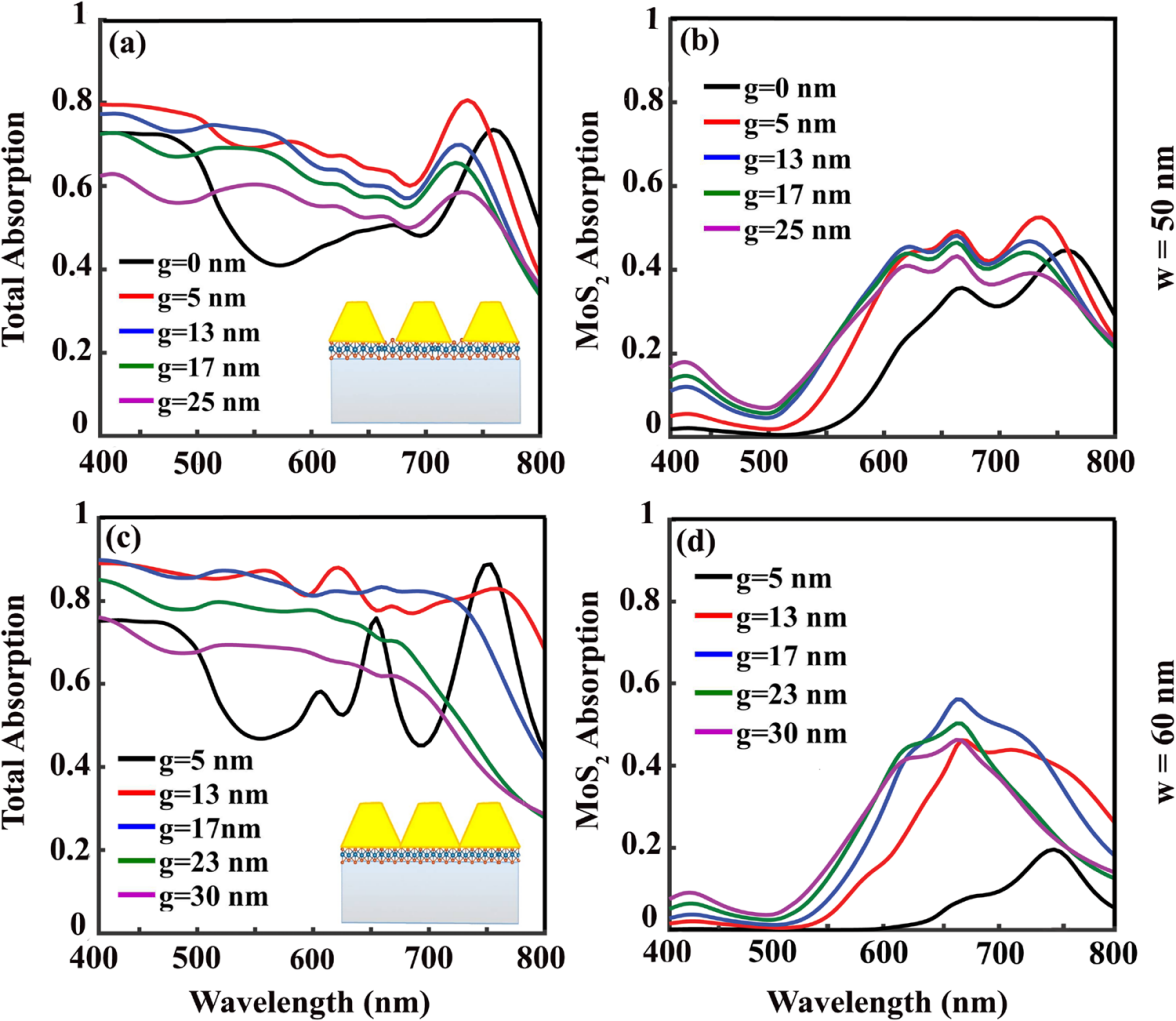
(**a**)/(**c**) Total and (**b**)/(**d**) MoS_2_ absorption spectrum as a function of *g*, when the gold grating parameters are set to $h=110$ nm, $p=60$ nm, $w=50$ nm/ $w=60$ nm.

## Absorption enhancement by inclined gold gratings

In a try to increase the absorption bandwidth and value simultaneously, we introduce another variation to the gold grating’s structure as the ribbon’s inclination. The gold grating’s inclination effect is studied by sweeping over *g* values from 0 to 25 nm in the grating structure with $p=60$ nm and $w=50$ nm that leads to the total and MoS_2_ absorptions presented in Fig. 4a and b, respectively. Compared with the absorption of the non-inclined grating, $g=0$, with the maximum total absorption of 73%, the absorption reaches 80% at its maximum value with $g=5$ nm (Fig. 4a). Interestingly, in this structure within the entire range of the visible spectrum, minimum absorption is not less than 605 which leads to a 25% variation in the total absorption relative to the maximum absorption of 80%. An increase in MoS_2_ absorption peak value from 45% in case of $g=0$ to 52% within the structure with $g=5$ nm is obvious in Fig. 4b. The full width at half maximum (FWHM) of the MoS_2_ absorption reaches 215 nm in case of $g=5$ nm with this maximum absorption value of 52%. This way, by introducing a slight inclination of $g=5$ nm to the gratings, both total and MoS_2_ absorption values and their bandwidths increase significantly.

To continue our survey in increasing total and MoS_2_ absorption, we examine the case of inclined gratings with $p=w=60$ nm. It has worth of mentioning that, by considering the grating’s inclination, setting the grating width equal to the grating period does not lead to a uniform gold layer extension on MoS_2_; instead, the gold ribbons are periodically serrated geometries as it is shown in the inset of Fig. 4c. With $g=13$ nm not only maximum absorption reaches 88% but also surprisingly the bandwidth of the absorption spectrum extends from $\lambda =400$ nm to $\lambda =780$ nm. In this wide wavelength range which covers almost the entire visible spectrum, one can not find any single wavelength with light absorption less than 77%, 12% variation relative to the maximum value, which is a great achievement (Fig. 4c). Setting $g=17$ nm in the grating with $p=w=60$ nm keeps the total absorption in the wavelength range of $\lambda =400$ nm to $\lambda =720$ nm as a smooth curve with the fixed absorption value of 81% (Fig. 4c). Maximum light absorption of 56% in MoS_2_ is also occurs within the structure with $g=17$ nm, which is a significant value (Fig. 4d).

This way, according to the structure’s application, both cases of $g=13$ nm and $g=17$ nm can be considered as wide-band absorbers with high efficiencies. If we were looking for a structure with efficient and wide-band absorption of MoS_2_, inclined gratings with $p\ne w$, e.g. the studied case of $p=60$ nm and $w=50$ nm with $g=5$ nm and absorption FWHM of 215 nm, would be more practical (Fig. 4b).

**Figure 5:**
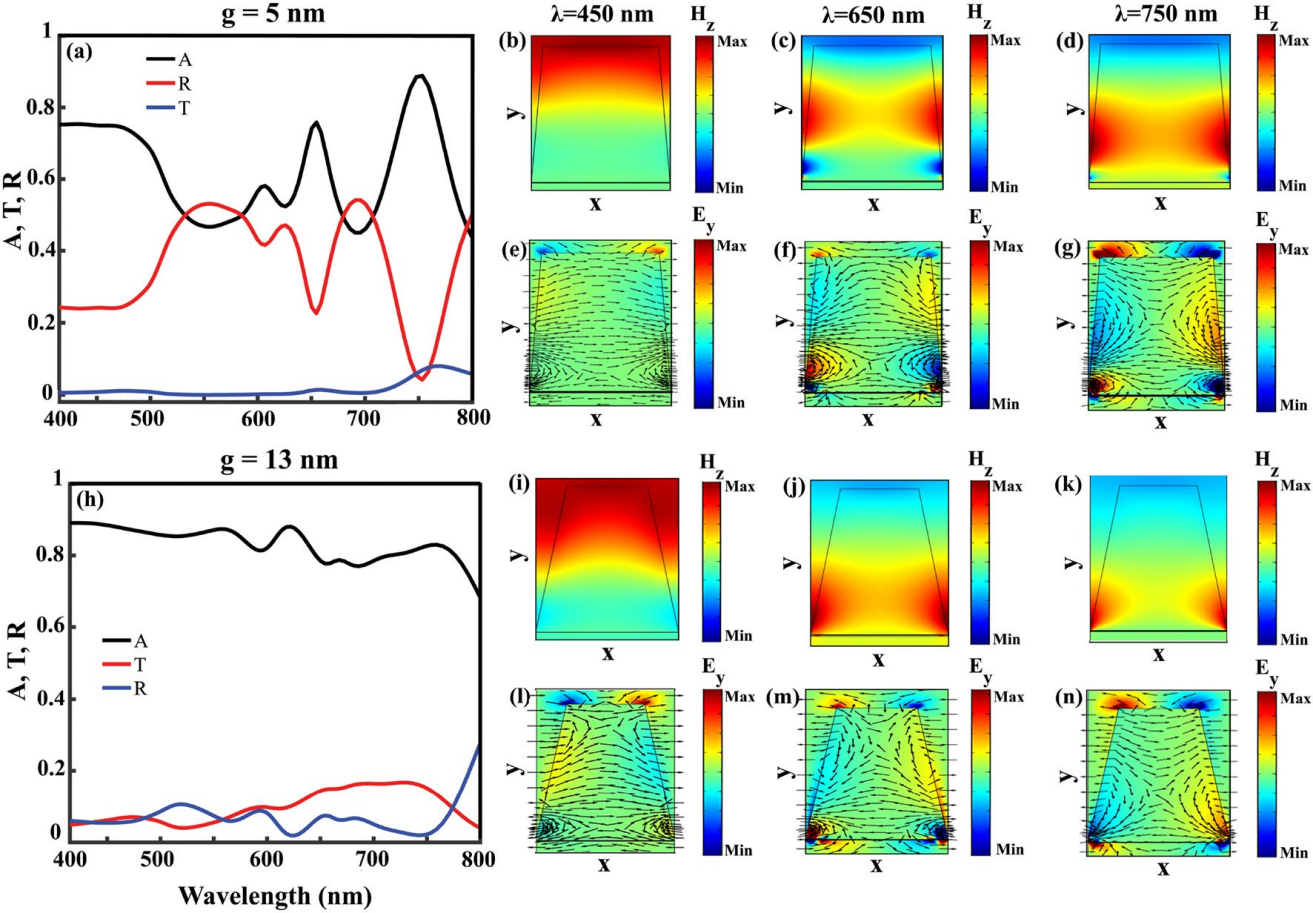
Reflection (R), transmission (T), and absorption (A) spectrum of the structure with $p=w=60$ nm, (**a**) $g=5$ nm and (**h**) $g=13$ nm. At $\lambda =450$ nm/ $\lambda =650$ nm/ $\lambda =750$ nm, (**b**) and (**i**)/(**c**) and (**j**)/(**d**) and (**k**) show the $H_{z}$, and (**e**) and (**l**)/(**f**) and (**m**)/(**g**) and (**n**) illustrate the $E_{y}$ field distributions in the structure with $g=5$ nm and $g=13$ nm, respectively.

Among the studied structures, we concentrate on $p=w=60$ nm with two cases of $g=5$ nm in which the individual absorption peaks are well-separated and $g=13$ nm with high absorption values in the entire range of visible spectrum and investigate the physics behind these absorptions in details. In Fig. 5a we start our study with the case of $g=5$ nm with including the absorption (black line), reflection (red line), and transmission (blue line) of the structure. Three wavelengths are selected for detailed examination: $\lambda =450$ nm with absorption of 75% is depicted from a wide range absorption, which extends from $\lambda =400$ nm to $\lambda =470$ nm, and two absorption peak wavelengths $\lambda =650$ nm and $\lambda =750$ nm with 76% and 89% absorptions, respectively. It can be seen that, at the wavelengths with maximum light absorption, reflection dips can be recognized in Fig. 5a. With the TM polarization of the incident light, the interesting $H-$ and $E-$field components for further studies are $H_{z}$ and $E_{y}$. Looking at the $H_{z}$ distribution at $\lambda =450$ nm in Fig. 5b with its related $E_{y}$ at Fig. 5e, reminds us the field distribution of the structure with $p=60$ nm, $w=50$ nm, with no grating inclination, $g=0$ (Fig. 3c). This way, excitation of “reflection” modes are responsible for the observed high absorption values at the wavelength range of $\lambda =400$ nm to $\lambda =470$ nm.

At $\lambda =650$ nm, Fig. 5c, and $\lambda =750$ nm,Fig. 5d, the $H_{z}$ distributions are illustrative for LSP excitation, similar to Fig. 3d, with the trapped light in the grating gaps. To find the source of this light trap, looking at the $E_{y}$ distribution is informative (Fig. 5f and g). For the ease of understanding, both arrow type and colored distribution of $E_{y}$ are included. In both Fig. 5f and g, the vortex-like $E_{y}$ arrows, together with the blue (negative minimum value) to red (positive maximum value) color changes in $E_{y}$ from one sharp edge to the other proves the resonant excitation of LSPs in the form of magnetic dipoles.

**Figure 6:**
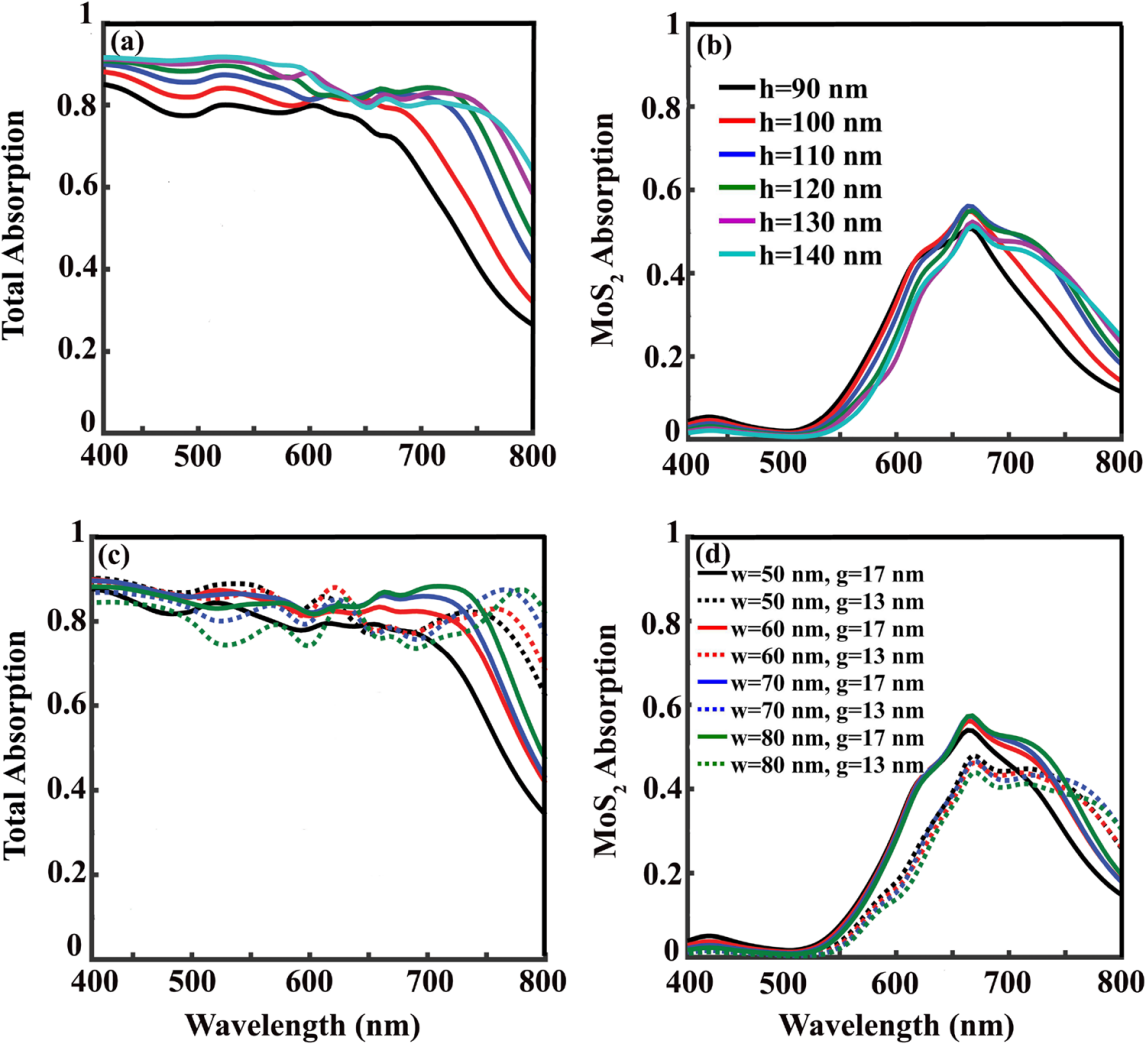
(**a**) Total and (**b**) MoS_2_ absorption spectrum as a function of grating height ,h, when $p=w=60$ nm and $g=17$ nm. (**c**) Total and (**d**) MoS_2_ absorption spectrum as a function of *p* for two different values of *g* as $g=13$ nm and $g=17$ nm with $h=110$ nm and $w=p$.

At this point, by looking at the absorption, reflection, transmission, and also $E_{y}$ and $H_{z}$ field distributions of our studied wide-band absorber with $p=w=60$ nm and $g=13$ nm in Fig. 5h–n, excitation of similar resonant modes to the studied case of $g=5$ nm can be recognized. The studied $H_{z}$ and $E_{y}$ field distributions are included in Fig. 5i and l at $\lambda =450$ nm, Fig. 5j and m at $\lambda =650$ nm, and Fig. 5k and n at $\lambda =750$ nm, respectively. Compared to the case of $g=5$ nm, stronger localization and light trap in the grating gaps occur in the case of $g=13$ nm, which can be seen by comparing Fig. 5j and k with $g=13$ nm with Fig. 5c and d with $g=5$ nm. This stronger light localization happens together with guiding the excited LSPs close to the MoS_2_ layer. Taking a look at Fig. 4d and comparing the MoS_2_ absorption of the two cases of $g=5$ nm with maximum absorption of 20% and $g=13$ nm with maximum 46%, confirms well the accumulation of the incoming energy close to the MoS_2_ layer in case of $g=13$ nm. By reminding the thickness of MoS_2_ monolayer, 0.62 nm, this amount of light absorption in it is surprising. Thanks to the excitation of LSPs which prepared the proper condition to use the potential of this material in our designed absorber. Height of the gold gratings, *h*, is another deterministic parameter in the absorption value which is investigated in Fig. 6. In a grating structure with $p=w=60$ nm with $g=17$ nm, starting from $h=90$ nm and increasing it with steps of 10 nm to $h=140$ nm shows that further height increment from $h=110$ nm doesn’t change total (Fig. 6a) and MoS_2_ (Fig. 6b) absorptions. This way, there would be no point in increasing the grating height more than 110 nm. Further investigations, not presented in here, show that irrespective to the value of *p*, within the structures with $p=w$, both absorption value and bandwidth reach their optimum values when $h=110$ nm, and $g=13$ or 17 nm.

In Fig. 6c and d total and MoS_2_ absorptions of structures with different periods, *p*, are studied when *w* is set to be equal to *p* and $h=110$ nm for two different values of *g* as $g=13$ nm (dotted lines) and $g=17$ nm (solid lines). It can be deduced that increasing the grating’s *p* more than 60 nm doesn’t change the absorption value and bandwidth significantly. It is of course a relief in designing such an absorber that if the period accuracy violates, it will not affect the absorber functionality too much. In the studied cases, with $g=13$ nm, the bandwidth of the total absorption with values above 77% is wider (up to $\lambda =780$ nm in case of $p=60$ nm), while with $g=17$ nm the bandwidth is less (up to $\lambda =720$ nm in case of $p=60$ nm) but with higher values of absorption of 81%. MoS_2_ absorption of 56% is the maximum value that reached in gratings with $g=17$ nm and *p* values of 60, 70, and 80 nm.

At this point, it has worth mentioning that compared with the metamaterial-based MoS_2_ absorbers which are mostly consist of complicated three dimensional unit cells^51–53^ , without loss of efficiency or bandwidth, our proposed absorber has a two-dimensional design with easy-to-fabricate unit cell.

## Conclusion

Within the design that is proposed in this paper, broadband absorption of MoS_2_-based absorber that covers the entire visible spectrum, from 400 nm to 780 nm, with absorption efficiencies above 77% and maximum of 88% is presented. Within the shorter wavelengths, 400 nm to 470 nm, the incident light mainly absorbs in the form of named “reflection” mode inside the gold grating, while in the longer wavelengths MoS_2_ absorption plays an essential role. Enhanced light absorption in MoS_2_ occurs by guiding the excited localized surface plasmons (LSPs) in the empty space between two subsequent ribbon of the gold grating toward the MoS_2_. Guiding the LSPs close to the MoS_2_ is done by inclining the gold gratings. By introducing an inclination value of $g=17$ nm to the gratings with $p=w=60$ nm, 56% of the incoming light absorbs in MoS_2_ monolayer. The presented method of using TMDCs with metallic structures in designing absorbers is a promising way to achieve miniaturized broadband and high efficient absorbers which are applicable in designing photovoltaic cells, photodetectors, and modulators.

## Data Availability

All data needed to evaluate the conclusions in the paper are present in the paper. Additional data related to this paper may be requested from the authors.
